# Pharmacokinetic Models of Tafenoquine: Insights for Optimal Malaria Treatment Strategies

**DOI:** 10.3390/pharmaceutics16091124

**Published:** 2024-08-26

**Authors:** Luisa Oliveira Santos, Izabel Almeida Alves, Francine Johansson Azeredo

**Affiliations:** 1 Laboratory of Pharmacokinetics and Pharmacometrics, Faculty of Pharmacy, Federal University of Bahia, Salvador 40170-110, Brazil; santos.luisa@ufba.br (L.O.S.);; 2 Center for Pharmacometrics & System Pharmacology, Department of Pharmaceutics, College of Pharmacy, University of Florida, Orlando, FL 32827, USA

**Keywords:** tafenoquine, population pharmacokinetics, pharmacokinetics/pharmacodynamics

## Abstract

Tafenoquine (TQ) is a new 8-aminoquinoline antimalarial drug developed by the US Army for *Plasmodium vivax* malaria treatment. Modeling and simulation are essential tools for drug development and improving rationality in pharmacotherapy, and different modeling approaches are used. This study aims to summarize and explore the pharmacokinetic (PK) models available for tafenoquine in the literature. An integrative methodology was used to collect and review published data. Fifteen articles were identified using three modeling approaches: non-compartmental analysis (NCA), population pharmacokinetic analysis (popPK), and pharmacokinetic/pharmacodynamic analysis (PK/PD). An NCA was mainly used to describe the PK profile of TQ and to compare its PK profile alone to those obtained in association with other drugs. PopPK was used to assess TQ population PK parameters, covariates’ impact, and dose selection. PK/PD helped understand the relationship between TQ concentrations, some adverse events common for 8-aminoquilones, and the efficacy assessment for *Plasmodium falciparum*. In summary, pharmacokinetic models were widely used during TQ development. However, there is still a need for different modeling approaches to support further therapeutic questions, such as treatment for special populations and potential drug–drug interactions.

## 1. Introduction

Malaria is a preventable and treatable infectious disease that spreads worldwide and is caused by protozoan parasites of the genus *Plasmodium*, which are transmitted through the bites of infected female *Anopheles* mosquitoes [1]. The most common and prevalent species infecting humans are *P. falciparum* and *P. vivax*, and clinical disease manifestation depends on the level of immunity [1]. Malaria can be presented as uncomplicated malaria, in which the patient presents non-specific symptoms, like fever and nausea, or severe malaria, in which one or more severe symptoms occur without an alternative cause, like severe anemia and renal impairment [1].

In 2022, there were 249 million estimated cases distributed in 85 countries. Despite the large number of cases globally, the Americas demonstrated a decline in malaria cases in the World Health Organization (WHO) 2023 malaria report [2]. In this region, most of the cases are due to *P. vivax*, which is one of the few species of *Plasmodium*-forming hypnozoites, the dormant parasite stage in the liver that can cause relapse weeks to years after the primary infection [1,2]. Treatment choice depends on the species of the infection and disease severity, but overall, the treatment for both uncomplicated and severe malaria is an artemisinin-based combination therapy (ACT) [1,3].

The treatment objective for *P. vivax* infections is to cure the acute blood stage and clear hypnozoites from the liver, making important therapeutic agents active against both stages [1]. The WHO recommendation is an association of an ACT for blood-stage malaria and 14 days of primaquine for hypnozoites, a prolonged treatment course with studies pointing to a compliance issue [1,4,5]. Tafenoquine (TQ) is a new 8-aminoquinoline antimalarial drug developed by the US Army and GlaxoSmithKline Pharmaceuticals (GSK) for treating relapsing *P. vivax* malaria and malaria prophylaxis. TQ is a prodrug that needs CYP2D6 metabolism, is active against both blood and liver stages of malaria, and has promising characteristics for therapy, such as a high distribution volume and a long half-life, which makes a single-dose regimen possible, providing a new perspective for a *P. vivax* radical cure [6,7,8,9]. However, as a new drug, there are some questions about its behavior that remain unclear, such as potential drug–drug interactions with transporters mentioned in labels based on in vitro studies.

Modeling and simulation (M&S) are essential tools for drug development and improving rationality in pharmacotherapy [10]. M&S have been used for the characterization of drug candidates, and different modeling methods are used, from population pharmacokinetic (popPK) modeling and pharmacokinetics/pharmacodynamics (PK/PD) to non-compartmental analyses [11]. Regulatory agencies, such as the U.S. Food and Drug Administration (FDA) and the European Medicines Agency (EMA), provide guidelines about M&S reports for drug registration. TQ development has used different M&S methods for drug characterization, dose selection, and optimization for special populations. Thus, this study aims to summarize and explore the pharmacokinetic (PK) models available for tafenoquine in the literature.

## 2. Methodology

An integrative methodology was used to collect and review published data on the PK modeling of tafenoquine, a new antimalarial drug. Five stages produced an integrative review: problem identification, literature search, data evaluation, data analysis, and data presentation [12].

### 2.1. Research Question

The research question that guided this integrative review was “What pharmacokinetic models are available for tafenoquine?” so that it would be possible to access the knowledge development of this drug.

### 2.2. Search Strategy

The search strategy included four databases: PubMed, Web of Science, Scopus^®^, and Embase. These databases were searched until May 2024, and the search terms included “tafenoquine”, “WR 238605”, and “pharmacokinetics”. These search terms were selected based on a previous literature search. A manual search of reference lists of relevant articles related to the search theme was also performed.

### 2.3. Study Selection

Inclusion and exclusion criteria for this review were identified based on the research question. Titles and abstracts were screened based on the following inclusion criteria: involving PK modeling and model description. Exclusion criteria were studies that did not report PK model development and results. All types of studies that met these criteria were included. Two screening stages were used to select the articles. The first screening stage included the review of all titles and abstracts based on the inclusion criteria. Articles that passed the first stage continued to a full-text screening, where all full-text manuscripts were screened to ensure they followed the inclusion criteria. Two investigators screened titles and abstracts for inclusion. Discrepancies were resolved through discussion and consensus.

### 2.4. Data Collection and Data Analysis

Data from the online query were screened, and replicated articles were eliminated. Titles and abstracts were analyzed to select only those articles in which PK modeling was performed. Then, the full-text articles were screened to include studies that reported model development and results. Data extraction was performed using a form on Excel^®^ 2019 version 1808 and included information on author, year, study purpose, design, methodology, and findings.

### 2.5. Data Presentation

The data extracted from the selected studies were grouped based on their modeling method so they could be compared in terms of development and the results presented.

## 3. Results and Discussion

**Figure 1:**
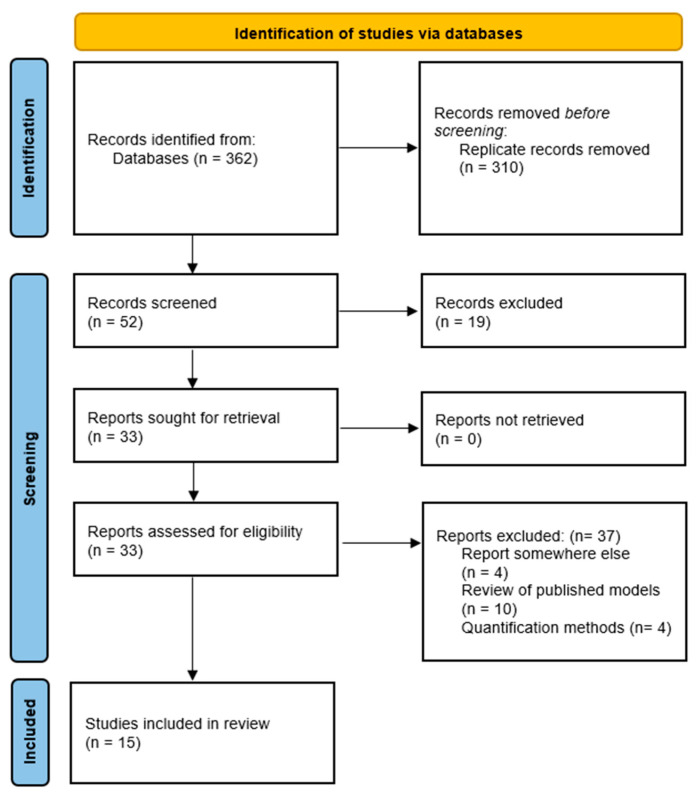
PRISMA flow diagram [13].

A total of 362 articles were identified in the databases, and then, titles and abstracts were screened. Three hundred ten replicates were eliminated, and of the fifty-two remaining titles and abstracts, screening and full-text screening were performed; the study selection flow chart is detailed in Figure 1.

All the articles selected were quantitative studies. They were organized based on their pharmacokinetic evaluation approach in three sections: non-compartmental analysis (NCA), population pharmacokinetic analysis (popPK), and pharmacokinetic/pharmacodynamic analysis (PK/PD). In summary, the articles found were published during the drug development, and since TQ was first approved in 2019, there are few new articles about it, with most of the studies from the early 2000s. Table 1 illustrates the data collected.

### 3.1. Non-Compartmental Analysis

An NCA is a model-independent method because it can describe drug PK without a compartment assumption [28,29]. However, an NCA requires a few assumptions, such as the PK needing to be linear and the terminal phase needing to be log-linear [28,30]. The technique used in an NCA involves the trapezoidal rule for the measurement of the area under the plasma concentration–time curve (AUC) based on statistical moment theory as well as other mathematical calculations to determine parameters such as the half-life, mean residence time (MRT), and λ [28,29]. An NCA is mainly used to estimate PK parameters and describe the drug PK profile since this method does not account for interindividual variability, which can be critical in clinical practice. [28,31]. Therefore, researchers often complement an NCA with more sophisticated modeling approaches, such as population pharmacokinetic (popPK) or pharmacokinetic/pharmacodynamic (PK/PD) modeling, to gain a comprehensive understanding of drug behavior.

Eight articles used an NCA, with the main objectives of these studies being to describe the PK profile of the drug and evaluate drug interactions [7,14,15,16,17,18,19,20]. Three of these were preclinical studies, and five were clinical trials. The results found in these studies are summarized in Table 2.

One of the animal studies assessed the impact of the different CYP2D6 metabolism statuses on tafenoquine PK in mice; an NCA was used to compare the PK profile of tafenoquine in CYP2D knockout (KO) mice—which represent poor metabolizers—and wild-type CYP2D (WT) mice—which represent extensive metabolizers [14]. KO mice presented a longer half-life (t1/2), a higher total area under the curve (AUC_total_), and decreased apparent clearance (CL) than the WT strain, as observed in Table 2. Liver PK presented the same behavior, with the KO strain exhibiting a higher C_max_ (85.8 ng/mL for KO versus 29.3 ng/mL for WT), a higher AUC_total_ (10,317.7 h·μg/mL for KO versus 3447.9 h·μg/mL for WT), and lower CL than the WT (2.0 mL/h/kg for KO versus 6.0 mL/h/kg for WT) [14]. This analysis contributes evidence that CYP2D metabolizer status alters TQ PK in mice.

Li and collaborators compared tafenoquine and primaquine (PQ) using an NCA to determine the initial PK parameters of TQ and PQ in the plasma and liver of mice after intravenous and oral administration [15]. Comparing the oral PK of both drugs, TQ has a higher C_max_ and AUC_total_, a longer elimination half-life, and slower CL than PQ [15]. Pharmacokinetic parameters estimated using liver concentrations (liver PK) were similar to those observed in plasma, and these results point to TQ as a more potent schizonticidal drug than PQ because of its bioavailability, prolonged elimination, and accumulation in the blood, which increases drug exposure [15]. Both of these studies accessed the TQ profile in mice to obtain a better human perspective on the drug distribution profile in the liver.

A preclinical study was performed with a Rhesus macaque model to evaluate a potential pharmacokinetic interaction of ivermectin (IVM), TQ, and chloroquine (CQ) for mass drug administration (MDA) [17]. A comparison between the PK profile of TQ alone and co-administered with IVM or IVM plus CQ was used to assess the potential interaction. The TQ AUC_total_ was higher following co-administration with IVM plus CQ compared to TQ alone and IVM co-administration (Table 2). Co-administration also alters the elimination of the half-life and CL, with a slower elimination when TQ is associated with IVM plus CQ [17]. The results demonstrated that TQ exposure increases with CQ co-administration rather than IVM, which requires further studies since the number of subjects in this trial (n = 4) was limited. This macaque trial presented a higher AUC_total_ and C_max_ than what was observed in mice.

Pharmacokinetic research using animal models yields essential preliminary data on a drug’s ADME attributes, playing a critical role in the preparation for human clinical trials. These investigations contribute to determining appropriate dosages and refining treatment protocols, thereby streamlining the drug development process. Nonetheless, animals’ distinct physiological and metabolic profiles compared to humans may result in predictions that do not translate accurately, leading to potential risks associated with dosing efficacy and safety. Furthermore, the inability of animal studies to fully replicate human disease states, coupled with ethical and regulatory complexities, may impede the progress of research. The excessive dependence on animal data also risks neglecting the advancement of alternative methods that are more directly applicable to humans, such as in vitro techniques or computer-based models [32,33].

The other five studies using an NCA were clinical trials, and three were associated with different modeling approaches (popPK and PK/PD). The study that did not associate an NCA with another modeling approach was an assessment of potential drug interactions between TQ and two other first-line artemisinin-based combination therapies (ACTs)—dihydroartemisin–piperaquine (DHA-PQP) and artemether–lumefantrine (AL) [16]. The PK parameters from the NCA were used to compare the PK profile of TQ alone and co-administered with DHA-PQP or AL, and a modest increase was observed in the TQ C_max_, AUC_total_, and half-life when co-administered with DHA-PQP; AL co-administration showed no effect on the C_max_, AUC_total_, and half-life of TQ, meaning that overall the parameters showed no important changes and confirmed a lack of clinically significant drug interactions of TQ and these ACTs [16].

Miller and collaborators also performed a drug interaction assessment and reported their results in a 90% confidence interval range. They used an NCA to compare PK parameters of TQ alone and with CQ, which presents a slight increase in the TQ C_max_ of 38% on day 2 of co-administration plus CQ, and the AUC_total_ fell within the 0.8–1.25 equivalence interval, which indicates no PK interactions. However, the co-administration did not show relevant changes in the drug’s PK profile [18]. The three selected articles assessing interactions used an NCA; this can be explained because an NCA is a good approach to evaluating drug–drug interactions, as it allows for a comparison across PK parameters in different situations and gives a better understanding of their impact on parameters.

Regarding the other three studies, an NCA was used to describe the PK profile of the drug [7,19,20]. One of these was a first-in-human (FIH) with eighty volunteers receiving 600 mg of TQ that identified a long absorption phase, with linearity over the doses from 4 to 600 mg [7]. Barber and collaborators assessed four dose regimens of TQ (200, 300, 400, and 600 mg) to define the minimum single dose to clear asexual blood-stage *P. falciparum*. The results point to a dose-related increase in TQ exposure, which includes the C_max_ and AUC_total_ (Table 2), and the 400 and 600 mg doses showed rapid parasite clearance and exhibited therapeutic potential [20]. Absorption was slow based on the T_max_, and the elimination half-life was long (13 days), as observed in the FIH, meaning that the TQ profile is consistent even though the FIH was published in 1998 using quantification and modeling tools available at that time [7,20].

Finally, the last article used an NCA to analyze the PK profile of TQ in three dose regimens (300, 600, and 1200 mg) and their potential to cause QT interval prolongation [19]. The exact proportionality with increased exposure and dose that was observed in the other two studies was reported here by observing the C_max_ and AUC_total_ (Table 2) [19]. However, this study did not calculate the half-life of TQ using an NCA because of its long half-life and the study limitation regarding the short sampling collection time of 72 h post-dose [19].

Overall, the PK parameters of TQ are cohesive among the human studies, with a linear PK profile. Barber and collaborators found higher exposure to TQ than what was observed by Green and collaborators in 2014, although they worked with a smaller number of subjects (*n* = 12) and limited samples, which can bias the results. TQ presented a high Vd, and some of these studies argue about its binding to red blood cells, but there are previous articles describing how aminoquinolines accumulate in lysosomes in tissues [34]. The longer half-life and slow clearance are the most relevant characteristics of TQ within the studies, as it is a new therapy for *P. vivax* infection with a single dose as its differential. 

### 3.2. Population Pharmacokinetic Analysis

PopPK modeling attempts to predict concentration values for each individual and the population while quantifying the variability within the population, which can arise from interindividual and residual variability [28,35]. It can also identify covariates that impact drug variability, such as demographic characteristics. Population modeling is a crucial M&S method in drug development because it can analyze sparse data collected at unscheduled times, assess interindividual variability, and identify contributing factors. Therefore, popPK results often provide important recommendations in drug labels for special populations, for example [35,36,37]. 

Seven articles used popPK, with the primary goals being to describe the population PK profiles, assess the impact of different covariates, and select doses for adults and pediatric patients [7,22,23,24,25,26,27]. Six of these were clinical trials—four in adults and two in pediatric patients—and one used previously published data in adults. Three adult clinical trial studies reported a better data fit using a one-compartment model with first-order absorption and elimination without a lag time in absorption, and covariates like age and weight did not significantly impact the model [7,22,23]. The other four studies had a better fit in a two-compartment model with first-order absorption and elimination, including an absorption lag time [24,25,26,27]. These findings are summarized in Table 3.

One that had a better fit with one compartment was an FiH accessing TQ profile with an NCA and population analysis. Interindividual variability was calculated for Vd, CL, and ka with 26%, 44.6%, and 57.8%, respectively, which is a moderate indicating variable response in this study population, especially in the absorption constant, which is expected for water-insoluble drugs, and TQ presents this characteristic [7,38]. The other two studies that reported a one-compartment model were assessing TQ as a malaria prophylactic alternative and used different error models to check residual variability. Edstein and collaborators used an exponential error model (C_ij_ = C_pred,ij_ × e^εij^), and Charles and collaborators used a combined proportional and additive error model (C_ij_ = C_pred,ij_ ×(1 + ε_1,ij_) + ε_2,ij_), where C_ij_ is the ith observed concentration for the jth individual, C_pred,ij_ is the plasma TQ concentration predicted, and ε_ij_ is a randomly distributed variable with zero mean and variance of σ² that are attributed to different variabilities [36,37].

Both studies checked the impact of covariates by adding them to the base model and observing the change in the objective function values (OFVs); if there was a decrease in the OFV, the covariate was included in the model [24,25]. The parameters found in these articles are summarized in Table 3, and both studies did not report the need to use covariates such as age, weight, and ethnicity. These articles emphasized that the study population was limited and homogenous, indicating a need for further studies with a broader population. This might explain the difference between these and the two-compartment models, in which some covariates were included.

Articles reporting a two-compartment model included demographics of subjects from different countries (i.e., Brazil, Peru, Thailand) with a broader range of ages and weights. Residual variability was described by an additive error model with log-transformed data, which reflect an exponential error model, and covariates were assessed using goodness-of-fit between observed and predicted data and a drop in the OFV [23]. However, the relationship between weight and PK parameters was evaluated using an allometric relationship, and other covariates were tested with a power model centered using the median value for the sample (P_ij_ = θ_pop,j_ × (cov_ind_/cov_med_) ^θcov^ and P_ij_ = θ_pop,j_ × θ_cov_^cat^), where P_ij_ is the estimate of parameter j in the ith individual, θ_pop,j_ is the population value for the parameter, cov_ind_ is the individual covariate value, cov_med_ is the median covariate value for the population, θ_cov_ is a parameter that expresses the covariate effect, and cat is a categorical variable [23].

Thakkar and collaborators reported an allometrically scaled body weight as a covariate on apparent oral clearance (CL/F), apparent inter-compartmental clearance (Q/F), the apparent volume of distribution for the central compartment (V_2_/F), and the apparent volume of distribution for peripheral compartments (V_3_/F) as well as the inclusion of formulation type on the systemic bioavailability (F) and absorption constant rate (K_a_) and the health status on the apparent volume of distribution [23]. Formulation types included tablets and capsules, and the PK parameters identified in the model are presented in Table 3 [23]. The impact of health status is discussed because the analysis pointed out that malaria patients may suffer from dehydration, leading to a lower volume of distribution than healthy volunteers [23]. On the other hand, Tenero and collaborators did not describe the impact of covariates, only the model structure, but it appears this will be addressed in a future article [22]. This study evaluated the relationship between dose, drug exposure, and response, helping the dose selection [22].

A population model developed in adults was used as the base model for the pediatric trials using a two-compartment and allometric body weight scaling for clearance and the volume of distribution; interindividual variability was evaluated for CL and Vd; and an additive error with log-transformed data—which reflects an exponential residual error model—was used to describe the residual variability [26,27]. The formulation was assessed as a covariate on drug bioavailability as a power function like the adult model, and model development was guided by OFVs, goodness-of-fit, and visual predictive checks (VPCs). The model framework worked well for children and helped predict appropriate doses for children from 6 months to 15 years weighing 5 kg or more [26,27]. The PK parameters are described in Table 3.

The predicted doses for the pediatric population ranged broadly from 5 to 15 mg/kg, achievable with the approved 300 mg dose for adults, which means that there is no need for adjustment for the pediatric population. However, the studies had limitations related to the need for a comparator group, like a placebo group, and the short follow-up period, which could not capture *P. vivax* relapses, making them cautious about extrapolating these results to a broader population. Despite those limitations, these studies suggest a new perspective on *P. vivax* malaria treatment for children considering that TQ is limited to adult treatment. In summary, the popPK studies assessed the feasibility of TQ as a malaria prophylactic therapy for travelers, assessed the lack of clinically relevant differences in exposure across tablet and capsule formulations, and helped with the dose selection for phase III clinical trials, and they are making it possible to evaluate the utility of the approved dose of 300 mg for pediatric patients. These major findings regarding optimal malaria treatment reinforce the usage of TQ as a new antimalarial drug.

### 3.3. Pharmacokinetic/Pharmacodynamic Analysis

PK/PD modeling quantitatively estimates dose–response relationships, including therapeutic or toxic responses, and helps optimize the dosing regimen, which is especially important for antimicrobial agents [39,40]. These models can also be used to associate preclinical data with human data to understand the framework better, and studies are pointing to the use of translational PK/PD models to reduce animal use and improve sampling and study design [28,41,42]. Five articles used PK/PD modeling to assess the relationship between TQ concentrations and various types of adverse events (AE) related to the drug, exposure, or efficacy endpoint [18,19,20,21,22]. Four articles were clinical trials, and one was an animal assay. The findings are summarized in Table 4.

Two studies evaluated the relationship between TQ concentrations and QT interval prolongation using TQ alone and the association of TQ plus CQ [18,19]. Increases in QT intervals were observed in the combined therapy group with a maximal change from a baseline of 30.7 milliseconds (ms), similar to that of CQ alone of 26.7 milliseconds. Still, the results did not indicate an association between TQ concentrations and change-from-baseline QT intervals [18]. This study also assessed TQ and CQ co-administration and QT prolongation and did not find any effect of the drug’s association more than what was previously reported for CQ alone [18,43,44].

The effect of supratherapeutic (1200 mg) and clinical (300 and 600 mg) TQ doses on QTcF interval was within the upper bounds of the 90% confidence interval (CI) for QTcF variation (<10 milliseconds) [19]. Using nonlinear mixed effects, the PK/PD model is described in Table 4, and its shallow slope (0.5 ms/μgml^−1^) describing the relationship between TQ concentrations and QTcF interval demonstrated no effect of TQ on QTcF interval prolongation, a known class effect of quinolone antimalarial drugs [18,19,43]. Moxifloxacin was used as a positive control for QTcF alterations, with a high slope of 3.1 ms/μgml^−1^.

Brueckner and collaborators assessed the relation between TQ concentrations and methemoglobin levels using a two-compartment model from NONMEM [21]. Methemoglobinemia, a condition characterized by increased methemoglobin (MHb) in the blood, is a common AE among 8-aminoquinolines. A two-compartment model best described the data, and the PK parameters were similar to those observed across all PK assessments of TQ; this is the oldest study retrieved, with a large distribution volume and slow clearance [21]. PD analysis suggests a sigmoid E_max_ model with an E_max_ of 31.3% MHb and EC_50_ of 596 ng/mL, with an average %MHb of 14%. Still, TQ seems to have a reduced potency to cause this compared to primaquine [45,46]. The model evaluated had some limitations related to sample size, although the model provided satisfactory estimations of plasma TQ concentrations and MHb levels and the times of these levels in a validation group of three additional dogs, which made it possible to use this model to guide the design of other animal studies to assess other questions regarding TQ behavior [21].

In addition, Barber and collaborators evaluated the association between TQ concentrations and parasite clearance for *P. falciparum* [20]. A mammillary physiological model comprising seven compartments with first-order absorption and linear elimination was used to describe the PK of TQ and its metabolite (5,6-orthoquinone tafenoquine), and the relationship between TQ concentrations in red blood cells and parasite clearance was represented by an E_max_ PK/PD model [20]. Interindividual variability had low precision and was fixed at 0.07 based on retrospective modeling, and the VPC demonstrated a good fit among observed and simulated data [20]. The model predicted a MIC of 74 ng/mL, and these results support that the parasite clearance of *P. falciparum* with TQ is feasible. Still, the required dose was 400 or 600 mg, which is high and makes this treatment alternative dependent upon prior G6PD screening to avoid the risk of hemolysis [20,47].

Lastly, a report about TQ exposure–response on *P. vivax* patients used a PK/PD model to characterize the relationship between a dose ranging from 50 to 100 to 300 to 600 mg and the relapse-free efficacy of TQ [22]. The base model estimates the probability of a clinical response, with exposure (AUC) as a predictor variable and other covariates being evaluated based on OFV drop [22]. A parametric time-to-event approach was used, where TQ exposure (AUC) affects the time to recurrence, and the recurrence was coded as an event [22]. The final model contained exposure as a continuous variable and region as statistically significant covariates; based on it, relapse-free odds at six months increased by 51% for an AUC above the median value of 54.5 μg*h/mL, as it increased approximately 3.6-fold in subjects from Thailand [22]. There was no difference in the AUC by country or race, and the observed efficacy between 300 and 600 mg TQ pointed to the 300 mg dose for further studies since this was a phase 2b study, which is the current dose recommendation for *P. vivax* infections.

Therefore, the PK/PD models were mainly used to evaluate the TQ relationship with common adverse effects of 8-aminoquinolines, especially the QT interval prolongation, showing no clinical effect and proving TQ safety. The oldest article retrieved was a PK/PD model for TQ concentrations and %MHb, which appears to have been used to design animal studies during the preclinical phase. Besides that, PK/PD models were responsible for assessing the exposure–response relationship and helping with dose selection for P. vivax infection, as discussed in the popPK section, in addition to confirming the potential use of TQ as a *P. falciparum* therapy.

### 3.4. Results Summary

**Figure 2:**
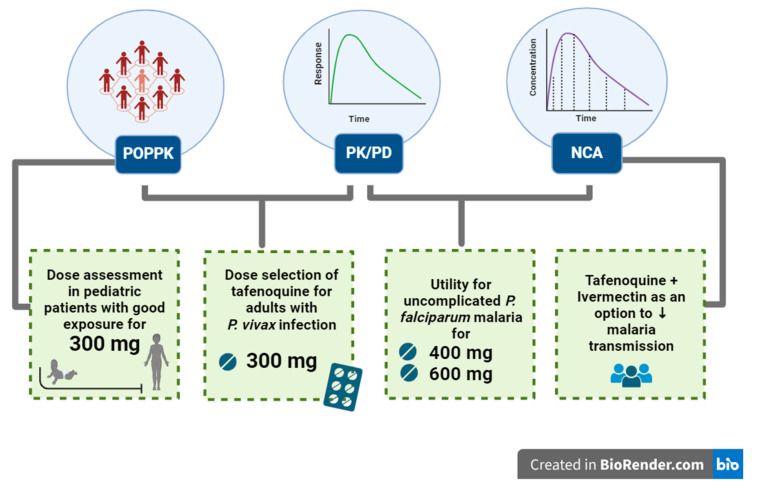
A summary of the main optimization findings.

The main insights for optimal malaria treatment are presented in Figure 2. These results are related to dose selection, with a TQ 300 mg dose being reported as the therapeutic dose for *P. vivax* malaria in adults and with good exposure in pediatric patients from 6 months to 15 years, although further studies are required in this population. The results also point to a new treatment perspective for *P. falciparum* uncomplicated malaria with 400 and 600 mg doses of TQ and also a new option for MDA or prophylaxis with TQ plus IVM to reduce malaria transmission.

Since integrative reviews can encounter several obstacles, including selection bias, diverse study designs, and challenges in synthesizing data, the reliability of conclusions may be affected by the quality of the included studies and subjective interpretations. This review had some limitations that should be considered, such as the small number of articles retrieved with the search strategy used, which may have compromised the breadth of the analysis, but this is not unexpected because TQ is relatively new to the market. Furthermore, the quality of the chosen articles and their strength for extrapolations are not homogenous, and many of the articles faced limitations related to small sample sizes and the lack of a control group. These factors can influence the robustness of their conclusions, and outdated studies may not reflect the accurate TQ profile. However, it was still possible to assess the current knowledge of TQ pharmacokinetic models and how they provide new perspectives on malaria treatment.

## 4. Conclusions

The findings of this review indicate that different empirical and mechanistic models have been developed for TQ, even though it is a new drug with few published articles. Most studies described the PK profile of TQ and assessed the safety and efficacy of the drug compared with PQ, the current WHO treatment recommendation for *P. vivax* malaria, and evaluated potential interactions with antimalarial drugs that could be associated with it during treatment, which seems reasonable since the major part of these studies were published during TQ development, answering this important question. The results of this review allowed for the identification of the published models of TQ, which include studies using NCA PK evaluation, popPK, and PK/PD models. This range of M&S methods supports the importance of PK modeling in drug development since the models can assist in appraising different pharmacological and therapeutic questions, from drug behavior and PK profiles to drug interactions, dose selection, and optimization for special populations. However, there are still some questions regarding TQ’s behavior without answers, such as confirming its utility in pediatric patients, the apparent interaction with organic cation transporter-2 (OCT2) and multidrug and toxin extrusion (MATE) substrates, and the potential for increased concentrations of these protein substrates based on in vitro data reported in the label.

## Figures and Tables

**Table 1:** A summary of the articles selected.

Authors	Type of Model	Patients/Animal Study Characteristics	Dosage Regimen	Outcomes
Voung, et al., 2015 [14]	NCA	7 healthy male mice KO and WT	20 mg/kg (IG)	The study demonstrated that decreased CYP2D metabolism affected the PK of TQ in mice, and a human study with poor metabolizers of the CYP2D6 phenotype is required.
Li, et al., 2014 [15]	NCA	3 healthy male mice	5 mg/kg (IV) and 20 mg/kg (IG) TQ	They found that TQ was 5-fold more potent than PQ as a liver schizonticide, with prolonged elimination, longer half-life, and increased drug exposure in plasma and the liver.
Green, et al., 2016 [16]	NCA	118 healthy adult volunteers	300 mg TQ p.o.; 120 mg/960 mg DHA-PQP p.o.; 80 mg/480 mg AL p.o.; TQ+DHA-PQP; TQ+AL	The study found no clinically significant drug interactions between TQ and the first-line ACTs, and there is no need for dose adjustment for co-administration.
Vanachayangkul, et al., 2024 [17]	NCA	4 healthy male macaques	1 mL/kg (NG) TQ, IVM and CQ	TQ exposure was elevated by co-administration of CQ but not IVM, which suggests that IVM and TQ combinations for MDAs or prophylaxis are a viable option.
Miller, et al., 2013 [18]	NCA and PK/PD	58 healthy adults volunteers	300 mg CQ p.o.; 900 mg TQ p.o.; CQ plus TQ	This study demonstrated no clinically significant pharmacokinetic or pharmacodynamic interactions between CQ and TQ in healthy subjects, with no changes in PK parameters or QT intervals.
Green, et al., 2014 [19]	NCA and PK/PD	260 healthy adult volunteers	300 and 600 TQ p.o.; 400 mg TQ p.o. (3 days); 400 mg MXF p.o.	This study found no effect of clinical doses of TQ (300 and 600 mg) and the supratherapeutic dose (1200 mg) on QTcF interval prolongation compared to placebo.
Barber, et al., 2023 [20]	NCA and PK/PD	12 healthy adult volunteers infected with *P. falciparum*	200, 300, 400 and 600 mg TQ p.o.	Single low dose (200–300 mg) of TQ was not effective at clearing asexual parasitemia. However, higher doses (400–600 mg) were effective, demonstrating that single-dose TQ exhibits potent *P. falciparum* blood-stage activity.
Brueckner and Fleckenstein, 1991 [21]	NCA and PK/PD	6 healthy male Beagles	Four daily doses of 6 mg/kg TQ p.o.	The PK/PD model provided a good estimate of plasma TQ concentrations and MHb levels as well as the times of their occurrences, and the model can be useful to guide the design of further animal studies and initial phase 1 human studies.
Brueckner, et al., 1998 [7]	NCA and popPK	80 healthy adults volunteers	4, 16, 36, 72, 100, 144, 192, 240, 250, 288, 300, 350, 400, 500 and 600 mg TQ p.o.	They observed gastrointestinal side effects at higher doses (300–600 mg), as well as linear PK. A one-compartment model with no impact of covariates best described the data.
Tenero, et al., 2015 [22]	popPK and PK/PD	180 patients with uncomplicated *P. vivax* mono-infection	600 mg CQ (day 1 and 2); 300 mg CQ (day 3); 50, 100, 300 or 600 mg TQ (day 1 or 2); 15 mg PQ (day 2–14)	The results of this phase 2b dose–response study showed that doses of 300 and 600 mg of TQ plus CQ had significantly improved relapse-free efficacy at 6 months compared with CQ alone. They also observed that the efficacy of PQ was lower than that of TQ at 300 and 600 mg. The 300 mg dose was proposed for further clinical studies.
Thakkar, et al., 2018 [23]	popPK	841 subjects collected from 5 studies (193 healthy adult volunteers and 648 patients)	50, 100, 300 and 600 mg TQ p.o.; 400 mg TQ p.o. (3 days)	The study found the final model to be a two-compartment model with allometric scaling for clearance and volume of distribution. No dose adjustment is needed based on other demographic characteristics, and this model found no difference in exposure across different formulations.
Edstein, et al., 2001 [24]	popPK	135 healthy adult volunteers	LD of 400 mg TQ p.o. (3 days); MD of 400 mg TQ p.o. monthly (5 months)	They observed a one-compartment model with first-order absorption and elimination. Covariates as age and weight did not have a significant impact on clearance and volume of distribution.
Charles, et al., 2007 [25]	popPK	490 healthy adult volunteers	LD of 200 mg TQ p.o. (3 days); MD of 200 mg TQ p.o. weekly (6 months)	The results showed a one-compartment model with first-order absorption and elimination, with no impact of covariates such as age and CL_CR_. The study supports a weekly dosing regimen for prophylaxis prolonged periods.
Bachhav, et al., 2022 [26]	popPK	59 pediatric patients with *P. vivax* malaria	100, 150, 200 and 300 mg TQ p.o.	The study demonstrated with a popPK model that microsampling may be an alternative sampling technique for PK studies because key exposure parameters, such as AUC_total_ and C_max_, are comparable across the venous sample model and the capillary microsampling model
Vélez, et al., 2022 [27]	popPK	60 pediatric patients with *P. vivax* malaria	100, 150, 200 and 300 mg TQ p.o.	A pediatric population pharmacokinetic model was developed, and they found a similar relative bioavailability between 50 mg dispersible tablets and 150 mg tablets. The 300 mg dose approved for adults demonstrated good exposure across all the weight and age bands.

Abbreviations are as follows: NCA—non-compartmental analysis; popPK—population pharmacokinetic modeling; PK/PD—pharmacokinetic/pharmacodynamic modeling; TQ—tafenoquine; KO—knockout; WT—wild type; IG—intragastric; IV—intravenous; p.o.—per os (oral administration); DHA-PQP—dihydroartemisinin-piperaquine; AL—artemether-lumefantrine; IVM—ivermectin; CQ—chloroquine; MXF—moxifloxacin; PQ—primaquine; LD—loading dose; MD—maintenance dose; QTcF—QT interval corrected for heart rate using Fridericia’s correction; CL_CR_—creatinine clearance; AUC_total_—total area under the concentration versus time curve; C_max_—peak concentration; PK—pharmacokinetics.

**Table 2:** Main information of NCA evaluation of tafenoquine.

Reference	Parameters					
	AUC_total_	C_max_	T_max_	t_1/2_	CL	Vd
Li et al., 2014 [15]	1.31 μg*h/mL (PQ)	0.53 μg/mL (PQ)	0.50 h (PQ)	1.84 h (PQ)	57.94 L/h/kg (PQ)	154.66 L/kg (PQ)
	139.18 μg*h/mL (TQ)	2.04 μg/mL (TQ)	11.33 h (TQ)	50.87 h (TQ)	0.15 L/h/kg (TQ)	10.60 L/kg (TQ)
Voung et al., 2015 [14]	85.6 μg*h/mL (WT)	1.2 μg/mL (WT)	5 h (WT)	53.8 h (WT)	6.0 mL/h/kg (WT)	18.5 L/kg (WT)
	123.2 μg*h/mL (KO)	1.4 μg/mL (KO)	10 h (KO)	72.4 h (KO)	2.0 mL/h/kg (KO)	19.9 L/kg (KO)
Vanachayangkul et al., 2024 [17]	3597 ng*h/mL (TQ)	67.4 ng/mL (TQ)	12 h (TQ, TQ+IVM, TQ+IVM+CQ)	45.8 h (TQ)	0.64 L/h/kg (TQ)	40.2 L/kg (TQ)
	3248 ng*h/mL (TQ+IVM)	56 ng/mL (TQ+IVM)		49.4 h (TQ+IVM)	0.75 L/h/kg (TQ+IVM)	49 L/kg (TQ+IVM)
	6508 ng*h/mL (TQ+IVM+CQ)	84.4 ng/mL (TQ+IVM+CQ)		63.2 (TQ+IVM+CQ)	0.33 L/h/kg (TQ+IVM+CQ)	29.7 L/kg (TQ+IVM+CQ)
Brueckner et al., 1998 [7]	n.a	n.a	13.8 h	325 h	6.1 L/h	2534 L
Miller et al., 2013 [18]	0.84–1.4 (TQ)	1.17–1.64 (TQ)	n.a	0.94–1.20 (TQ)	n.a	n.a
	0.84–1.18 (TQ+CQ)	0.74–1.08 (TQ+CQ)	n.a	0.78–1.12 (TQ+CQ)	n.a	n.a
Green et al., 2014 [19]	10,611 ng*h/mL (300 mg) #	186 ng/mL (300 mg)	15 h (300 mg)	n.a	n.a.	n.a
	22,986 ng*h/mL (600 mg) #	422 ng/mL (600 mg)	12 h (600 mg)		n.a	
	41,896 ng*h/mL (1200 mg) #	724 ng/mL (1200 mg)	12 h (1200 mg)		n.a	
Green et al., 2016 [16]	97,195.5 ng*h/mL (TQ)	199.6 ng/mL (TQ)	12.1 h (TQ)	375.2 h (TQ)	3 L/h (TQ)	n.a
	109,333.7 ng*h/mL (TQ+DHA-PQP)	274.7 ng/mL (TQ+DHA-PQP)	6 h (TQ+DHA-PQP)	483.9 h (TQ+DHA-PQP)	2.7 L/h (TQ+DHA-PQP)	
	102,328.4 ng*h/mL (TQ+AL)	208.4 ng/mL (TQ+AL)	12.1 h (TQ+AL)	396.5 h (TQ+AL)	2.9 L/h (TQ+AL)	
Barber et al., 2023 [20]	47,091 ng*h/mL (200 mg)	138.2 ng/mL (200 mg)	12.2 h (200 mg)	309.4 h (200 mg)	3.8 L/h (200 mg)	1708 L (200 mg)
	67,303 ng*h/mL (300 mg)	205.8 ng/mL (300 mg)	12 h (300 mg)	357.3 h (300 mg)	3.9 L/h (300 mg)	2003 L (300 mg)
	117,107 ng*h/mL (400 mg)	324.8 ng/mL (400 mg)	10 h (400 mg)	313.9 h (400 mg)	3.1 L/h (400 mg)	1379 L (400 mg)
	128,140 ng*h/mL (600 mg)	340.2 ng/mL (600 mg)	12.1 h (600 mg)	314.4 h (600 mg)	4.1 L/h (600 mg)	1863 L (600 mg)

# AUC_0-tlast_. Abbreviations are as follows: AUC_total_—total area under de concentration versus time curve; C_max_—maximum concentration; T_max_—time to C_max_; CL—clearance; Vd—volume of distribution; t_1/2—_terminal half-life; KO—knockout; WT—wild type; TQ—tafenoquine; DHA-PQP—dihydroartemisinin-piperaquine; AL—artemether-lumefantrine; IVM—ivermectin; CQ—chloroquine; PQ—primaquine; n.a—not applicable.

**Table 3:** Main information of popPK models of tafenoquine.

Reference	Model	Covariates	Parameters				
Brueckner et al., 1998 [7]	One-compartment model	n.a	Vd = 2550 L; CL = 4.71 L/h; ka = 0.391 h^−1^				
Edstein et al., 2001 [24]	One-compartment model	n.a	Vd = 1820 L; CL = 3.20 L/h; T_max_ = 8.6 h; ka = 0.694 h^−1^; t_1/2_ = 16.4 dias				
Charles et al., 2007 [25]	One-compartment model	n.a	Vd = 1110 L; CL = 3.02 L/h; T_max_ = 21.4 h; ka = 0.243 h^−1^; t_1/2_ = 12.7 dias				
Tenero et al., 2015 [22]	Two-compartment model with absorption lag time	n.a	n.a	n.a	n.a	n.a	n.a
Thakkar et al., 2018 [23]	Two-compartment model with absorption lag time	Weight was included in CL and Vd; formulation status was associated with ka and bioavailability; health status was included as a covariate on Vd	V2/F = 915 L; V3/F = 664 L CL/F = 2.96 L/h; Q/F = 5.09 L/h Ka = 0.252 h^−1^				
Bachhlav et al., 2022 [26]	Two-compartment model with absorption lag time	Weight was included in CL and Vd; formulation status was associated with ka and bioavailability; health status was included as a covariate on Vd	V2/F = 798 L; V3/F = 732 L CL/F = 3.4 L/h; Q/F = 6.33 L/h Ka = 0.253 h^−1^				
Vélez et al., 2022 [27]	Two-compartment model with absorption lag time	Weight was included in CL and Vd; formulation status was associated with ka and bioavailability; health status was included as a covariate on Vd	V2/F = 917 L; V3/F = 724 L CL/F = 3.67 L/h; Q/F = 5.94 L/h Ka = 0.253 h^−1^				

Abbreviations are as follows: Vd—the volume of distribution; CL—clearance; ka—absorption rate constant; T_max_—time to C_max_; t_1/2_—half-life; V2/F—the volume of distribution of first (central) compartment; V3/F—the volume of distribution of second (peripheral) compartment; CL/F—oral clearance; Q/F—inter-compartment clearance; n.a—not applicable.

**Table 4:** Main information of PK/PD models of tafenoquine.

Reference	Model	Parameters
Miller, et al., 2013 [18]	PK analysis was conducted using NCA, PD assessment used electrocardiographs, and PK/PD assessment was performed with graphical analyses	For TQ: AUC_total_ = 0.84–1.14 ng*h/mL; C_max_ = 1.17–1.64 ng/mL; t_1/2_ = 0.94–1.20 h For TQ+CQ: AUC_total_ = 0.84–1.18 ng*h/mL; C_max_ = 0.74–1.08 ng/mL; t_1/2_ = 0.78–1.12 h
Green et al., 2014 [19]	Population analysis was used to establish the PK/PD relationship between TQ concentrations and the QTcF interval. It included the covariates sex and race describing the baseline, 2 cosine functions (8-h cycle and 24-h cycle), and 2 slope terms	For 300 mg: AUC_total_ = 10,611 ng*h/mL; C_max_ = 186 ng/mL; T_max_ = 15 h For 600 mg: AUC_total_ = 22,986 ng*h/mL; C_max_ = 422 ng/mL; T_max_ = 12 h For 1200 mg: AUC_total_ = 41,896 ng*h/mL; C_max_ = 724 ng/mL; T_max_ = 12 h
Tenero et al., 2015 [22]	The exposure–response model used nonlinear mixed-effects and the recurrence of *P. vivax* infection was modeled using a time-to-event approach where drug exposure affects the time to a recurrent episode	n.a
Barber et al., 2023 [20]	PK/PD model used a nonlinear mixed-effects and individual parameters from a population model as regression parameters	For 200 mg: AUC_total_ = 47,091 ng*h/mL; C_max_ = 138.2 ng/mL; T_max_ = 12.2 h; t_1/2_ = 309.4 h; CL = 3.8 L/h; Vd = 1708 L For 300 mg: AUC_total_ = 67,303 ng*h/mL; C_max_ = 205.8 ng/mL; T_max_ = 12 h; t_1/2_ = 357.3 h; CL = 3.9 L/h; Vd = 2003 L For 400 mg: AUC_total_ = 117,107 ng*h/mL; C_max_ = 324.8 ng/mL; T_max_ = 10 h; t_1/2_ = 313.9 h; CL = 3.1 L/h; Vd = 1379 L For 600 mg: AUC_total_ = 128,140 ng*h/mL; C_max_ = 340.2 ng/mL; T_max_ = 12.1 h; t_1/2_ = 314.4 h; CL = 4.1 L/h; Vd = 1863 L
Brueckner and Fleckenstein, 1991 [21]	Nonlinear least-squares regression was used for the PK/PD model with two-compartments, first-order absorption and elimination, linked to an effect compartment; the PD model used a sigmoid Emax to describe the %MHb	AUC_total_ = 311,097 ng*h/mL; Vd = 18.5 L/kg; CL = 83 mL/h/kg

Abbreviations are as follows: AUC_total_—total area under the concentration versus time curve; C_max_—maximum concentration; t_1/2_—half-life; T_max_—time to C_max_; NCA—non-compartmental analysis; TQ—tafenoquine; QTcF—QT interval corrected for heart rate using Fridericia’s correction; %MHb—percentage of methemoglobin; n.a—not applicable.
